# Proinsulin C-peptide is a major source of HLA-DQ8 restricted hybrid insulin peptides recognized by human islet-infiltrating CD4^+^ T cells

**DOI:** 10.1093/pnasnexus/pgae491

**Published:** 2024-11-01

**Authors:** Pushpak Bhattacharjee, Miha Pakusch, Matthew Lacorcia, Eleonora Tresoldi, Alan F Rubin, Abby Foster, Laura King, Chris Y Chiu, Thomas W H Kay, John A Karas, Fergus J Cameron, Stuart I Mannering

**Affiliations:** Immunology and Diabetes Unit, St. Vincent's Institute of Medical Research, 9 Princes St, Fitzroy, VIC 3065, Australia; Immunology and Diabetes Unit, St. Vincent's Institute of Medical Research, 9 Princes St, Fitzroy, VIC 3065, Australia; Immunology and Diabetes Unit, St. Vincent's Institute of Medical Research, 9 Princes St, Fitzroy, VIC 3065, Australia; Immunology and Diabetes Unit, St. Vincent's Institute of Medical Research, 9 Princes St, Fitzroy, VIC 3065, Australia; Bioinformatics Division, Walter and Eliza Hall Institute of Medical Research, Parkville, Melbourne, VIC 3052, Australia; Department of Medical Biology, University of Melbourne, Parkville, Melbourne, VIC 3010, Australia; Immunology and Diabetes Unit, St. Vincent's Institute of Medical Research, 9 Princes St, Fitzroy, VIC 3065, Australia; Immunology and Diabetes Unit, St. Vincent's Institute of Medical Research, 9 Princes St, Fitzroy, VIC 3065, Australia; Immunology and Diabetes Unit, St. Vincent's Institute of Medical Research, 9 Princes St, Fitzroy, VIC 3065, Australia; Immunology and Diabetes Unit, St. Vincent's Institute of Medical Research, 9 Princes St, Fitzroy, VIC 3065, Australia; School of Chemistry, University of Melbourne, Parkville, Melbourne, VIC 3010, Australia; Department of Endocrinology and Diabetes, Royal Children's Hospital, Parkville, Melbourne, VIC 3052, Australia; Murdoch Children's Research Institute, Parkville, Melbourne, VIC 3052, Australia; Department of Paediatrics, University of Melbourne, Parkville, Melbourne, VIC 3010, Australia; Immunology and Diabetes Unit, St. Vincent's Institute of Medical Research, 9 Princes St, Fitzroy, VIC 3065, Australia; Murdoch Children's Research Institute, Parkville, Melbourne, VIC 3052, Australia; Department of Paediatrics, University of Melbourne, Parkville, Melbourne, VIC 3010, Australia; Department of Medicine, University of Melbourne, St. Vincent's Hospital, Fitzroy, VIC 3065, Australia

**Keywords:** type 1 diabetes, hybrid insulin peptides, islet-infiltrating CD4^+^ T cells, neoepitopes

## Abstract

Type 1 diabetes (T1D) is an autoimmune disease that develops when T cells destroy the insulin-producing beta cells that reside in the pancreatic islets. Immune cells, including T cells, infiltrate the islets and gradually destroy the beta cells. Human islet-infiltrating CD4^+^ T cells recognize peptide epitopes derived from proinsulin, particularly C-peptide. Hybrid insulin peptides (HIPs) are neoepitopes formed by the fusion of two peptides derived from beta cell granule proteins and are known to be the targets of pathogenic CD4^+^ T cells in the non-obese diabetic (NOD) mouse and human islet-infiltrating CD4^+^ T cells. Proinsulin is widely recognized as a central antigen in T1D, but its role in forming HIPs is unclear. We developed a method to functionally screen TCRs derived from human islet-infiltrating CD4^+^ T cells and applied this to the identification of new proinsulin-derived HIPs. We generated a library of 4,488 candidate HIPs formed by fusion of proinsulin fragments and predicted to bind to HLA-DQ8. This library was screened against 109 islet-infiltrating CD4^+^ T cell receptors (TCRs) isolated from four organ donors who had T1D. We identified 13 unique HIPs recognized by nine different TCRs from two organ donors. HIP-specific T cell avatars responded specifically to a peptide extract from human islets. These new HIPs predominantly stimulated CD4^+^ T cell proliferation in peripheral blood mononuclear cells from individuals with T1D in contrast to HLA-matched controls. This is the first unbiased functional, islet-infiltrating T cell based, screen to identify proinsulin-derived HIPs. It has revealed many new HIPs and a central role of proinsulin C-peptide in their formation.

**Significance Statement**

Type 1 diabetes (T1D) is an autoimmune disease caused by T cells destroying the pancreatic insulin-producing beta cells. The targets, known as antigens, seen by disease-promoting T cells are poorly understood. Hybrid insulin peptides (HIPs) are a new class of antigen recognized by T cells that cause T1D in a mouse model. In humans, very few HIPs recognized by human islet-infiltrating T cells are known. We show that HIPs derived from proinsulin are recognized by human islet-infiltrating T cells from T1D donors and describe 13 new HIPs formed by fusion of fragments of proinsulin. This work shows that all HIPs recognized by islet-infiltrating T cells contain fragments of C-peptide. This underscores C-peptide's role as an autoantigen in human T1D.

## Introduction

Type-1 diabetes (T1D) is a chronic autoimmune disease caused by the T cell-mediated destruction of the pancreatic, insulin-producing, beta cells (1). This leads to insulin deficiency and dysregulation of glucose metabolism (2). Genetic risk of developing T1D is strongly associated with the HLA class II, specifically the haplotypes: HLA-DR3-DQ2 (HLA-DRB1*03:01-DQA*05:01-DQB*02:01) and HLA-DR4-DQ8 (HLA-DRB1*04:01; DQA*03:01, DQB*03:02) (3). Of these HLA-DR4-DQ8, haplotypes give the highest risk of developing T1D (3, 4).

Based on the genetic associations outlined above, CD4^+^ T cells that recognize beta cell antigens presented by HLA molecules encoded by the haplotypes HLA-DR4-DQ8 and/or HLA-DR3-DQ2 are associated with autoimmune beta cell destruction (5–7). Furthermore, insulin and its precursor proinsulin have emerged as important targets of autoimmune responses in T1D (8, 9). Specifically, C-peptide, which is excised from proinsulin, has emerged as major antigenic target of human islet-infiltrating (5, 7) and peripheral blood CD4^+^ T cells (10). We developed methods to isolate and characterized viable CD4^+^ T cell clones from the pancreatic islets of T1D organ donors (5). We (5), and others (6, 7, 11, 12), have shown that islet-infiltrating CD4^+^ T cells are strongly implicated in the immune pathogenesis of T1D. In addition to their presence at the site of autoimmune beta cell destruction, T cells isolated from the pancreatic islets of individuals who had T1D are specific for epitopes derived from insulin and proinsulin, and they are restricted by T1D-associated HLA allomorphs, particularly HLA-DQ8.

Why beta cells become the targets of T cell-mediated immune destruction is currently unknown. One attractive hypothesis is that CD4^+^ T cell responses may be directed against beta cell-specific neoepitopes which could explain why beta cells are not protected by mechanisms of T cell tolerance (13, 14). CD4^+^ T cell responses to several neoepitopes, including hybrid insulin peptides (HIPs) (15) have been associated with human T1D (14, 16–18). HIPs are formed by the fusion of two peptide fragments within the beta cell granules. CD4^+^ T cells specific for HIPs are pathogenic in the non-obese diabetic (NOD) mouse model of human T1D (15). For example, the T cell clone isolated from a diabetic NOD mouse with, BDC2.5, which is specific for a HIP formed by the fusion of C-peptide and chromogranin-A (ChgA) causes diabetes when transferred into NOD mice (15, 19).

CD4^+^ T cell responses to HIPs are increasingly implicated in the immune pathogenesis of human T1D. We (15) and others (11, 20) have shown that HIP-specific CD4^+^ T cells infiltrate the pancreatic islets of organ donors who had T1D. The presence of HIP-specific, HLA-DQ8 restricted CD4^+^ T cells within the pancreatic islets of individuals who had T1D strongly suggests that they play a causative role in the development of T1D. Studies using peripheral blood mononuclear cells (PBMCs) from individuals with, and without, T1D revealed that many of the original 12 HIPs identified by Delong et al. (15) were capable of stimulating CD4^+^ T cell responses in PBMC from individuals with T1D (21, 22). Arribas-Layton et al. (23) used HLA-DR4 tetramers and peptide binding assays to identify six HLA-DR4 (DRB1*04:01) restricted HIPs recognized by peripheral blood derived CD4^+^ T cells from individuals with T1D. Responses to HIPs arise early in the immune pathogenesis of T1D. CD4^+^ T cell responses to HIPs have been reported to be detectable in the peripheral blood prior to the onset of clinical T1D (24). Autoantibodies specific for extended HIP sequences formed by the fusion of fragments of proinsulin with IAPP2 (islet amyloid polypeptide) peptides were detected in the serum of individuals before insulin autoantibodies could be detected (25).

Despite their clear relevance to the immune pathogenesis of human T1D, few HIPs have been identified and their immunobiology remains poorly understood. Studying CD4^+^ T cell responses to HIPs faces two related challenges (26). First, since HIPs are formed by the fusion of two peptide fragments there is a very large number of candidate HIPs that can potentially be generated (26) making it very difficult to identify new HIPs. Second, it is difficult to link a CD4^+^ T cell response against a HIP to the pathogenesis of human T1D for ethical reasons. To address these challenges, we devised a high-throughput functional screening assay to identify proinsulin-derived HIPs. More specifically, to accommodate the large number of potential HIPs, we established an *Escherichia coli* library of candidate, proinsulin-derived, HLA-DQ8-binding HIPs. To ensure that we identified HIPs relevant to the immune pathogenesis of human T1D, we isolated human islet-infiltrating CD4^+^ T cells, sequenced their T cell receptors (TCR) genes, and then expressed these TCRs in a modified Jurkat line. The Jurkat lines, or T cell avatars, were used to screen the HIP library and identify HIPs recognized by TCRs derived from human islet-infiltrating CD4^+^ T cells. We reasoned that identifying HIPs formed from proinsulin, presented by HLA-DQ8, and recognized by human islet-infiltrating CD4^+^ T cells would be of relevance to the pathogenesis of human T1D. Using this approach, we identified 13 previously unknown HIPs recognized by human islet-infiltrating CD4^+^ T cells. Analysis of the sequences of these HIPs revealed that they all incorporated fragments of proinsulin C-peptide, indicating that C-peptide is an important contributor to T1D-associated human HIPs.

## Materials and methods

### Subjects and ethical approval

The isolation of pancreatic islets from deceased organ donors was approved by the St Vincent's Hospital Human Research Ethics Committee (approval no. SVH HREC-A 011/04). Ethical approval was given by St Vincent's Hospital HREC (approval numbers: 2022/PID05221 and HE135/08) and Southern Health/Royal Children's Hospital (approval number: 12185B). All participants provided written informed consent. Participants are without T1D or diagnosed according to American Diabetes Association criteria.

### Isolation and TCR sequencing of human islet-infiltrating CD4^+^ T cells

The isolation of pancreatic islets and the analysis of islet-infiltrating CD4^+^ T cells from Donor A has been reported previously (5). For the other donors the pancreatic islets were digested by Accutase (Merck) to obtain a single-cell suspension. These cells were stained with a cocktail of monoclonal antibodies (mAbs) conjugated with DNA-barcoded oligonucleotide that are specific for immune cell lineages (TotalSeq-C Human TBNK Cocktail, BioLegend) according to the manufacturer's protocol. At the same time, the cells were stained with the following fluorochrome-labeled mAbs: anti-CD45-AF488 (clone QA17A19) and anti-CD3-PE (clone HIT3a). Viable, propidium iodide negative, CD45^+^ and CD3^+^ cells were fluorescence-activated cell sorting (FACS) sorted using FACS Aria Fusion. The scRNA-seq, scTCR-seq and feature barcode libraries of the sorted islet-infiltrating T cells were prepared by using the 10× Genomics Chromium Single Cell Immune Profiling Solution Kit (5′ Gene Expression and V[D]J) as per the manufacture's protocol. The libraries were sequenced following Illumina's specifications using paired-end sequencing (2 × 150 bp) on Illumina NovaSeq system with a minimum of ∼5,000 reads/cell for V(D)J and feature barcode libraries. The scRNA-seq and scTCR-seq reads were aligned to the 10× prebuilt reference genome to GRCh38 (GRCh38-2020-A and vdj_GRCh38_alts_ensembl-5.0.0) and quantified using Cellranger multi pipeline (10× Genomics, version 6.0.1). Filtered gene count matrix from CellRanger was analyzed using R (version 4.1.0) and Seurat (version 4.0.5). Cell-specific filtering was performed by retaining cells with RNA features between 200 and 7,500, and less than 5% mitochondrial RNA. Centered log ratio transformation across cells by Seurat's function.

Data were used to normalize raw counts of feature barcode. Counts of gene and feature barcodes for CD4 and CD8 were used to identify CD4 single positive cells, further filtering excluded cells with: a single TRAV, a single TRBV, or multiple TRBV genes. This left cells with a single TRBV and one or two TRAV genes. TCR genes were cloned from the available T cell clones (Donor A), or all available TCR sequences (Donors I and J). For Donor K, the 52 most abundant TCR clonotypes were cloned (Table S5).

### Lentivirus production

For lentivirus-mediated gene transduction, variable regions of TCR genes were expressed and cloned into modified versions of pRRLSIN.cPPT.PGK-GFP.WPRE. The EGFP gene was excised by *Bam*HI/*Sal*I digestion and replaced by human TRAC or TRBC2 with either a *Pme*I (TRAC) or *Sbf*I (TRBC2) site at the 5′ end of the TCR constant regions. All islet-infiltrating TCR variable alpha (TRAV) and TCR beta (TRBV) genes were synthesized by Integrated DNA Technologies (IDT) as gene blocks and cloned into the modified pRRLSIN vectors by In-fusion cloning (Takara) according to the manufacturer's protocol. Plasmids were extracted from growing *E. coli* and the inserts were verified by Sanger sequencing. For transfection of HEK293T cells, TCR gene-containing plasmids were purified using Macherey-Nagel kits: NucleoBond Xtra Midi Plus (Midi prep), NucleoSpin Plasmid (Standard), or NucleoSpin Plasmid (Transfection Grade). All plasmid purifications were done according to the manufacturer's recommendations. HEK293T cells were transfected with the appropriate paired TCRs in pRRLSIN and the packaging plasmids: pMDLg/pRRE, pRSV-Rev, and pMD2.g using Lipofectamine 2000 (Invitrogen) as per manufacturer's instructions. After 7–8 h incubation at 37 °C, 5% CO_2_, the medium was changed. After a further 2.5 days' incubation, supernatant was collected and filtered through a 0.45 μm low protein binding filter. Lentivirus-containing supernatant was either used fresh, or frozen at −80 °C until required.

### Generation of T cell avatars

Starting with Jurkat E6.1, we generated a subline that was TCR deficient, CD4^+^ and had a luciferase reporter gene knocked immediately following the IL-2 promoter (Figure S1). Briefly, we used CRISPR/Cas9 to generate a variant of the human T cell line Jurkat that was deficient in TRAC, TRBC, and CD4. A NanoLuciferase (Promega) reporter was then knocked into the IL-2 locus and a clone that showed an activation induced luciferase response was isolated (Figure S1). This parental Jurkat line was then transduced with the TRAV and TRBV constructs that express the TRAV and TRBV genes that form the TCR expressed by human islet-infiltrating CD4^+^ T cells. Hence for each TCR two constructs were required, a TRAV and a TRBV. Transduced cells expressing CD3 and TCR were purified by flow cytometry or magnetic bead enrichment. Briefly, cells were resuspended in viral supernatant at 1–2 × 10^6^/ml with 5 μg/ml polybrene. Cells were centrifuged at 1,200 rpm (300 g) for 60 min at room temperature, then diluted 1:1 in fresh medium and incubated overnight at 37 °C, 5% CO_2_. Flow cytometry was performed on a Becton Dickinson LSR Fortessa to determine the proportion of CD3 and TCR expressing cells. The following antihuman mAbs were used for FACS staining: CD3-PE (UCHT1, BD Biosciences), and anti-TCRαβ-AF647 (IP26, BioLegend). Cells were stained with the appropriate mAb in phosphate buffered saline (PBS) 0.1% foetal bovine serum (FBS) for 20 min on ice, then washed twice. Dead cells were excluded by propidium iodide staining. Compensation settings were determined using single-color controls. TCR and CD3 expressing cells were purified by FACS or using REALease CD3 Microbead Kit (Miltenyi) following the manufacturer's instructions. All Jurkat T cell avatars used were >85% CD3^+^/TCR^+^.

### HIP library generation in silico

The protein sequence of human proinsulin was fragmented, in silico, into 12mer sequences with an overlap of 11 amino acid seq. Each proinsulin 12mers fragment sequence was fused with all other 12mers in both directions to obtain every possible HIP from proinsulin. This 24mer proinsulin HIP library was then filtered based on the predicted binding to HLA-DQ8 (HLA-DQA1*03:01; O3:02) using IEDB's HLA binding prediction algorithm (http://tools.iedb.org/mhcii/). Predicted epitopes that did not incorporate a junction, that were derived from an intact 12mer were excluded. The top 21% of DQ8 binder’s 12mer HIPs sequence were shortlisted. After excluding duplicate sequences, a total of 4,488 candidate HIPs were used for screening (Figure S2A).

### HIP library cloning and validation

The *E. coli* codon-optimized nucleotide sequences of these HIPs were obtained using the GenScript web platform. Each HIP oligo sequence was adjoined with the pKE1 BamH1/Asc1 digested cloning site homology sequence in both termini. The 4,488 candidate HIP oligo sequences containing the cloning homology sequences were divided into 136 pools, where each pool contained 33 unique HIPs (sequences available on request). These single-stranded HIP oligo pools were commercially synthesized by IDT. The oligo pools were amplified by PCR and cloned, in frame with glutathione-S-transferase (GST), into the expression vector pKE-1 (28), by In-fusion cloning. Briefly, the oligonucleotide pools were amplified and duplexed using CloneAmp HiFi PCR Premix (Takara) with HIP Pool forward and reverse primer (Table S1). The PCR products were purified using the Macherey-Nagel NucleoSpin column as per manufacturer protocol. The pKE-1 vector was linearized using BamH1/Asc1 (NEB) digestion. All amplified and purified HIP oligonucleotides were cloned into the linearized pKE1 vector by In-fusion cloning (Takara) according to the manufacturer's protocol. After ligation, the plasmids were transformed into *E coli* Stellar Competent Cells (Takara) and selected with ampicillin (50 μg/ml) and kanamycin (10 μg/ml) containing Luria Broth. The pool library plasmids were purified using Macherey-Nagel NucleoSpin kits. The pool HIP-expressing bacteria were generated by transforming the purified pool plasmid into the Rosetta strain of *E. coli* (Novagen). Four pool HIP-expressing Rosetta were combined to make a “super-pool”. In this way, 34 super-pool HIP library bacteria were generated from 136 HIP pools. All pools and super-pools of Rosetta were frozen in 70% glycerol stock for future use.

To validate the HIP library, a master pool containing all plasmids in the library was prepared. The master HIP Pool that contains 136 pools was amplified by PCR using Qiagen Taq polymerase with amplicon forward and reverse primer (listed in Table S1). The PCR products were gel purified using a Macherey-Nagel NucleoSpin Gel Clean-up kit. Amplicon indexing was done using IDT xGen cfDNA & FFPE DNA library preparation kit and sequencing was performed using a 150-cycle MiSeq platform in the AGRF facility. The abundance of the transcript was determined from the single-end library by using Kallisto (v0.46.1) to generate an index file, followed by using Kallisto quant with an estimated fragment length of 81 bp and an estimated standard deviation of 10 to obtain gene-level normalized transcript per million (TPM).

### Using T cell avatars to identify novel HIPs

The HIP super-pool bacteria were cultured from frozen glycerol stock in LB media with antibiotics at 37 °C with agitation 210 rpm. The overnight HIP-expressing bacteria cultures were diluted to an OD_600_ of 0.06 and grown in 48-well plates in 200 μl LB medium with antibiotics until an OD_600_ of 0.5 was reached. Protein expression was induced by adding isopropyl β-D-thiogalactopyranoside (2.0 mM, Sigma). Incubation was continued for another 4 h at 37 °C in a shaking incubator. All bacteria cultures were then centrifuged at 3,000 rpm for 10 min and the bacterial pellet was resuspended into 300 μl of Roswell Park Memorial Institute (RPMI) 1640 media/5% FBS with gentamycin (Sigma Aldrich) (30 μg/ml) to prevent further bacterial growth. 10 μl of resuspended bacteria from each super pool were cultured with 30,000 autologous Epstein-Barr virus (EBV)-transformed B cells in a 96-well Nunclon Delta white microwell tissue culture plate. After culturing overnight, the T cell avatars expressing TCRs from human islet-infiltrating CD4^+^ T cells were added (10,000 cells/well). The culture was in a final volume of 150 μl of RPMI/5% human serum with gentamycin (30 μg/ml). After 22–24 h of coculture, the NanoLuciferase activity was measured using the Nano-Glo Luciferase assay (Promega) according to the manufacturer's instructions. Luminescence was measured on a plate reader (Enspire Perkin Elmer). Responses are reported as the Δluciferase, which is calculated by subtracting the average background (no antigen) luciferase reading measured in the negative control cultures from the response measured in the other treatments.

HIPs that stimulated the T cell avatars were identified by subdividing the pools and screening individual colonies. When a colony was identified that stimulated a T cell avatar the plasmid was extracted and sequenced by Sanger sequencing using the pKE1 sequencing primers (Table S1).

### Validation of T cell response with synthetic peptide and HLA-restriction

Peptides (Genscript) were dissolved in dimethyl sulfoxide (DMSO) to 5.0 mM, aliquoted and stored at −80 °C. A full list of peptides is shown in Table S2. The CD4^+^ T cell avatars' responses to peptides were measured as described above; except synthetic peptides were used as antigens, instead of *E. coli* and the antigen/EBV cells were not cultured overnight before the T cell avatars were added. A T cell's response to antigen was measured as luciferase activity using the Nano-Glo Luciferase assay system (Promega) according to the manufacturer's instructions (as above). The HLA-restriction of the HIP-specific CD4^+^ T cell clones was determined in a two-step process as described previously (10). First, monoclonal antibodies specific for HLA-DR (clone L243), HLA-DP (clone B7/21), and HLA-DQ (clone SPV-L3) were added to the peptide-stimulated T cell cultures to a final concentration of 1.0 or 5.0 μg/ml. Second, the HLA alleles were determined using a panel of HLA class II deficient T2 cells that were transfected with HLA-DQA and HLA-DQB alleles indicated in the figure legends. The T cell avatar's responses were measured as above. For HLA-DR alleles, DR3-DQ2 (IHW09022) or DR4-DQ8 (IHW09031) homozygous lines were compared with autologous EBV.

### Preparation of tissue extracts

Frozen pooled islet (400,000 IEQ) and spleen (4.0 g) samples from 8 or 5 healthy organ donors, respectively. Tissues were resuspended in 5.0 ml of 4 M guanidine thiocyanide/1% trifluroacetic acid (TFA) and sonicated on ice for 3.0 min. The resultant lysate was passed through 70.0 μM cell strainer (Corning) and centrifuged at 1,600 rpm for 5 min and the supernatant collected. C18 Sep-Pak cartridges (Classic Short cartridge, Waters), a reverse-phase chromatography-based method that allows peptides to extracted from cell lysates. These cartridges were used to enrich the peptides from the tissue lysate. The cartridges were prepared by washing with 3 ml of 99% methanol and 1.0% TFA, then washed with TFA/salt (0.5% TFA, 0.5% NaCl) then with 2.0 ml of methanol, TFA, water (30% methanol, 1% TFA in water). The 4.5 ml of cell lysate was loaded into C18 Sep-Pak cartridges. The peptides were eluted in one fraction with 6.0 mL methanol/water/TFA (80:19:1, v/v/v). The eluate was lyophilized to dryness and dissolved in 30 μl of DMSO. This material is referred to as “peptide extract”. To test for T cell avatar response, T cells and autologous EBV-transformed B cells (both at 10,000 cells/well) were cultured in a white 96-well plate (Nunclon Delta), with either a 1:200 dilution of cell lysate, or synthetic HIP peptide (1.0 μM). T cell avatar responses were measured as described above.

### The CFSE-based proliferation assay

The CFSE (5,6-carboxylfluorescein diacetate succinimidyl ester) proliferation assays were performed as described previously (35). Briefly, PBMC from T1D subjects or non-T1D HLA-DQ2 or -DQ8 matched donors labeled with 0.1 μM CFSE (Life Technologies, Carlsbad, CA, USA) were cultured either with: DMSO (0.02%) or HIPs (1.0 μM), or tetanus toxoid (10 LfU/ml). After 7 days of culture the cells were washed in PBS and stained on ice with antihuman CD4-AlexaFluor-647 (clone OKT4, prepared in house). CD4^+^ T cell proliferation was measured by determining the number of CD4^+^, CFSE^dim^ cells for every 5,000 CD4^+^ CFSE^bright^ cells. The results are presented as a CDI which is the ratio of the number of CD4^+^ cells that have proliferated in the presence of antigen:without antigen (47).

### Statistics and graphs

Graphs were plotted using Prism 10.0.3 and LogoPlots generated (using https://weblogo.berkeley.edu/logo.cgi) Graphing and statistical analysis was done using Prism 10.0.3. Statistical significance was determined using one-way ANOVA and corrected for multiple comparisons using Dunnett's test. Responses were compared using the Mann–Whitney test statistical significance was defined as *P* < 0.05 as shown in the figure legends.

## Results

### Generation of a panel of human islet-infiltrating CD4^+^ T cell avatars

Previously, we have shown that two human islet-infiltrating CD4^+^ T cell clones recognized a HIP formed by the fusion of C-peptide with IAPP2 (15). Given the importance of HIPs in human CD4^+^ T cell response associated with T1D, we set out to systematically identify new proinsulin-derived HIPs. We focused on proinsulin because there is considerable evidence that it is a central autoantigen in the pathogenesis of T1D (5, 8, 10, 27). Specifically, we wanted to identify novel HIPs, formed by the fusion of peptides derived from proinsulin, that were recognized by human islet-infiltrating CD4^+^ T cells. To achieve this, we generated and screened 109 T cell avatars (Figure S1). Each avatar expressed the TRAV and TRBV derived from a single CD4^+^ T cell isolated from one of four organ donors who had T1D. Two donors had had T1D for a shorter duration (3 or 4 years), while the other two had had T1D for a longer duration (13 and 19 years) (Table S3).

### Bacterial-based antigen screening identifies new HIPs

**Fig. 1.**
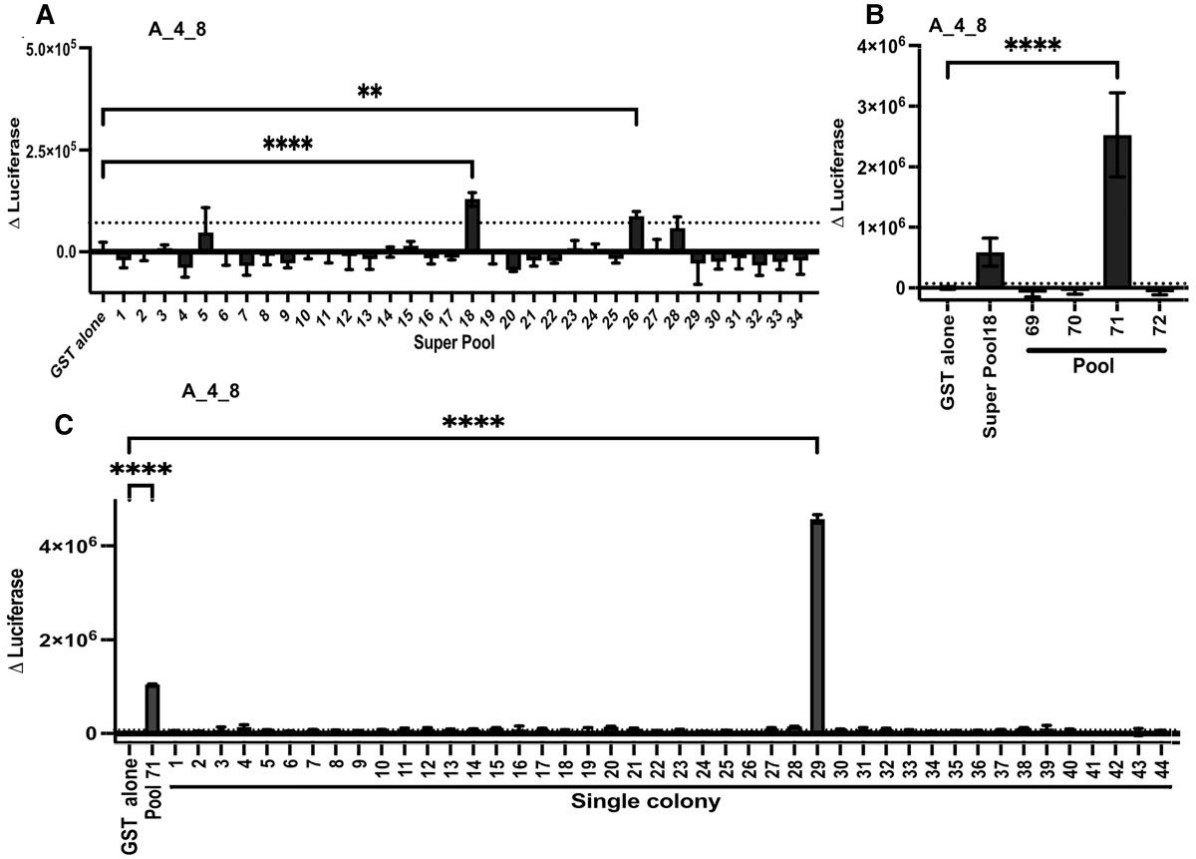
Functional screening of the candidate HIP library, expressed in *E. coli*, reveals new HIPs. An example of the screening of one T cell avatar (A_4_8) is shown. A) The HIP “super-pools” were tested against the T cell avatar A_4_8. B) HIP “super-pools” 18 and the four pools it comprises (pools: 69, 70, 71, and 72) were tested separately. C) Forty-four *E. coli* colonies isolated from HIP Pool 71 were tested against the A_4_8 T cell avatar. Antigen-specific responses were measured by luciferase activity in triplicate, except for the colony screening which was done in duplicate. The Δluciferase was calculated by subtracting the mean of the glutathione-S-transferase (GST) only plasmid-containing bacteria (GST alone) T cell avatar, antigen presenting cells treatments from all other readings. The bars represent the mean of Δluciferase ± SD. The dotted line is the GST alone treatment plus 2× SD represents the threshold for a positive response. Statistical significance was determined using one-way ANOVA and corrected for multiple comparisons using Dunnett statistical hypothesis testing and defined as *P < 0.05, **P < 0.01, ***P < 0.001, ****P < 0.0001.

We narrowed our search to HIPs that could form by the fusion of peptides derived from proinsulin (86 amino acids). We generated, in silico, a library of the 9,409 HIPs that could theoretically be formed by fusion of any two 12mer fragments of human proinsulin (Figure 1A). We then filtered these candidate HIPs to select unique sequences which were within the top 21% of predicted HLA-DQ8 (HLA-DQA1*03:01; DQB1*03:02) binding affinities. This led to a list of 4,488 12mer candidate, proinsulin-derived HIPs that predicted to bind strongly to HLA-DQ8. Hence, we screened 489,192 (109 × 4,488) combinations of T cell avatars and candidate HIPs. Oligonucleotides encoding these candidate HIPs were synthesized and cloned into an *E. coli* expression vector (28) and used to transform *E. coli.* Candidate HIPs that stimulated responses in the T cell avatars were identified using an optimized version (Table S4) of the method first described by Corput et al. (29). The library of 4,488 candidate HIPs was divided into 136 pools each comprising 34 candidate HIP sequences. To validate the library, a sample was sequenced, and the abundance of each target sequence was determined (Figure S2). These data show that >97.5% of the target sequences were present in the library at an abundance of >26 transcripts per million (TPM).

To identify novel proinsulin HIPs, we first optimized the workflow for detecting CD4^+^ T cell responses to pools of antigen-expressing *E. coli* (28) (results summarized in Table S4). Next, the candidate HIP library was screened in three steps. For the first round of screening, the 136 pools were combined into groups of four creating 34 “super-pools”. This made it feasible to screen all 4,488 candidate HIPs could be against 109 T cell avatars (Figure 1A). The results from screening one T cell avatar (A_4_8) at each of the three steps are shown in Figure 1. In this case, super-pool 18 stimulated a strong (ΔLuciferase **>** 1 × 10^4^ relative light units [RLU]) response from avatar A_4_8. Responses to some super-pools, particularly those that stimulated weak responses (ΔLuciferase **>** 1 × 10^4^ RLU), for example super-pool 26 (Figure 1A) could not be confirmed when we tested the component pools. The second step was to screen the avatars that responded to a super-pool against the same super-pool and each of the four component pools (Figure 1B). Avatar A_4_8 responded to a HIP expressed in pool 71. Finally, the T cell avatars that responded to each pool were tested against 40–80 individual *E. coli* clones isolated from the stimulating pool (Figure 1C). Colony 29 was the only colony screened that activated the TCR expressed in avatar A_4_8. The HIP sequences were determined by sequencing the plasmids isolated from the single stimulating colonies. After screening 109 avatars, we identified nine human islet-infiltrating T cell avatars, six from Donor A and three from Donor K, that responded to 13 unique candidate HIPs. These two donors were 3 or 4 y past diagnosis with stage 3 T1D. In contrast, no response to any candidate HIPs were detected in the T cell avatars isolated from the pancreatic islets of Donors I and J, who had T1D for 13, or 19 y, respectively (see Tables S3 and S5). T cell avatar responses to the HIP-expressing *E. coli* library were detected irrespective of the abundance of the HIP-expressing *E. coli* within the library (Figure S2B). Most epitopes identified were among the highest predicted binders to HLA-DQ8 (Table S11).

### Validating candidate HIP response

**Fig. 2.**
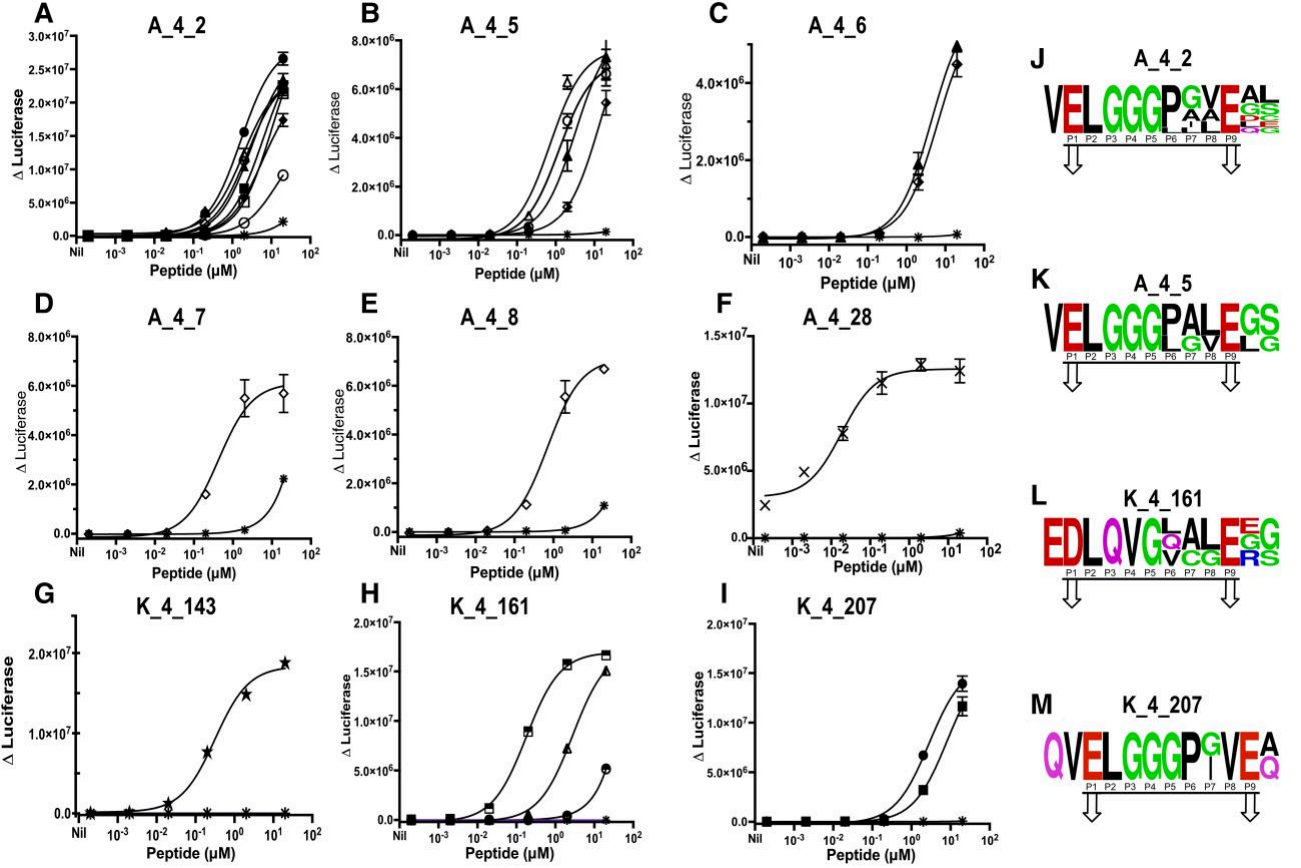
New HIPs are potent T cell agonists. The dose-response curves for the following T cell avatars are shown: A) A_4_2 tested against HIP-A (open circles), HIP-B (closed circles), HIP-C (open squares), HIP-D (closed squares), HIP-E (open triangles), HIP-F (closed triangles), HIP-G (open diamonds), HIP-H (closed diamonds), and C-peptide (star). B) T cell avatar A_4_5 was tested against HIP-A (open circle), HIP-E (open triangles), HIP-F (closed triangles), HIP-6 (half shaded diamond), and C-peptide (star). C) T cell avatar A_4_6 was tested against HIP-F (closed triangles), HIP-6 (half shaded diamond), and C-peptide (star); D) T cell avatar A_4_7 was tested against HIP-G (open diamonds) and C-peptide (star). E) T cell avatar A_4_8 was tested against HIP-G (open diamonds) and C-peptide (star). F) T cell avatar A_4_28 was tested against HIP-I (inverted triangles) and C-peptide (star). G) T cell avatar K_4_143 was tested against HIP-J (pentagram) and C-peptide (star). H) T cell avatar K_4_161 was tested against HIP-K (half shaded circles), HIP-L (half shaded squares), HIP-M (half shaded triangles), and C-peptide (star). I) T cell avatar K_4_207 was tested against HIP-B (closed circles), HIP-D (closed squares), and C-peptide (star). Antigen-specific responses were measured in triplicate by luciferase assay and represented by Δluciferase. ΔLuciferase was calculated by subtracting the mean of the “no peptide” treatment from the other treatments. Each point is the mean of triplicate values ± SD. One representative of at least two experiments is shown. J to M) The Logo plots for the multiple epitopes recognized by the T cell avatars: A_4_2 (J), A_4_5 (K), K_4_161 (L), and K_4_207 (M). The underline segment labeled showing putative core 9mer epitope is shown for each set of HIPs. The arrow showing the predicted HLA anchor residue for each set of HIP.

To confirm that the T cell avatars respond to the HIPs identified, we synthesized peptides with the same sequence of each candidate proinsulin HIP (Table S2). These peptides were used in titration experiments to measure their potency in stimulating the appropriate T cell avatars. In all cases, the response to HIPs expressed in *E. coli* were confirmed with synthetic peptides (Figure 2). These newly identified HIPs are referred to here as “HIP-A” to “HIP-M”. Some avatars recognized multiple HIPs. For example, the avatar expressing the TCR, A_4_2, responded to eight HIP sequences (Figure 2A), the most of any avatar. Two avatars (A_4_5 and K_4_161, Figure 2B and H) responded to three HIPs (HIPs-A, -E, -F and -K, -L, -M, respectively) and one avatar (K_4_207, Figure 2I) responded to two HIPs (HIPs-B and -D). Aligning the sequences in LogoPlots revealed that the C-terminal fragment of each HIP remained the same, but TCR recognition could tolerate several sequences on the N-terminal side of the HIPs (Figure 2J-M). The core 9mer epitope and the putative primary HLA anchor residues are also shown in the LogoPlots, based on the structural analysis of TCR–HIP interactions (30, 31). The non-HLA anchor residues may be TCR contacts, but these interactions can only be confidently defined by crystallography. Conversely, several HIPs stimulated more than one T cell avatar. HIP-B and HIP-D stimulated T cell avatars from Donor A (A_4_2) and Donor K (K_4_207) (Figure 2A and I and Table S9). Some HIPs (HIP-A, -B, -D, and -E), were able to stimulate two or three (HIP-F and -G) T cell avatars. We identified three TCRs from Donor A (A_4_2, A_4_7, and A_4_8) that were very sensitive to proinsulin HIPs, responding to as little as 100 nM of HIP peptide. Previously, we have reported that these TCRs also recognize epitopes derived from C-peptide (5), but when using T cell avatars in this work the responses to full-length C-peptide were very weak, even at the highest concentration (20 μM) tested (Figure 2A, D and E). Two other Donor A TCRs (A_4_5 and A_4_6) that we had previously reported to respond to a C-peptide-IAPP2 HIP (15) also responded with similar sensitivity to HIP-A, -E, -F, and -F, respectively (Figure 2B and C).Our *E. coli*-based screening method revealed HIPs that were weak TCR agonists, for example, HIP-K which is recognized by the avatar K_4_161 has an EC_50_ of >20.00 μM. In contrast, HIP-I was the most potent, it stimulated the avatar A_4_28 with an EC_50_ of 20 nM (Figure 2F and Table S9). Extended sequences from each “side” of the HIP did not stimulate the T cell avatars. For the left-hand side of each HIP 3 of 9 T cell avatars showed a weak response at the highest peptide concentration tested, 20 μM. No responses were detected to the extended right-hand side native peptides (Figure S3). We conclude that the *E. coli* screening method accurately identified new HIPs and most of these HIPs are potent activators of TCRs derived from human islet-infiltrating CD4^+^ T cells.

### Determining the HLA restriction of HIP-specific avatars

The HLA-restriction of the response to each HIP was determined by blocking TCR-mediated antigen recognition with HLA isotype-specific mAbs and then testing antigen-presenting cells expressing a single HLA class II allomorph. As expected, 11 of 13 (85%) HIP-specific T cell avatars were HLA-DQ8 restricted (Figure S4). However, two avatars (A_4_28 and K_4_143) were restricted by HLA-DR4 (Figure S4F and G). A summary of the islet-infiltrating T cell avatars' response to the new proinsulin HIPs is shown in Table S9. The responses to proinsulin-derived HIPs were all restricted by HLA allomorphs strongly associated with risk of developing T1D (32).

### HIP-specific T-cell avatars respond to islet extracts

**Fig. 3.**
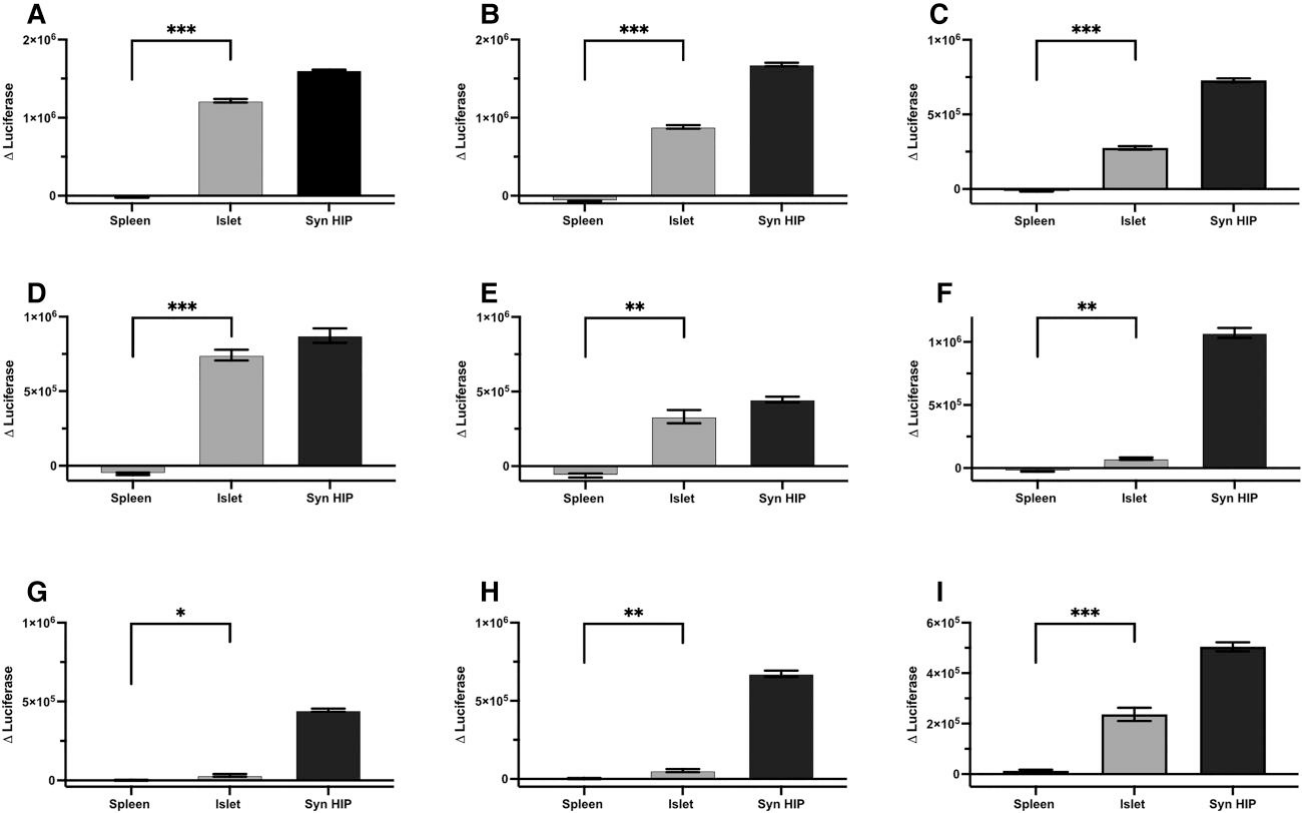
T cell avatars that recognize new HIPs also respond islet-derived peptides. The following T cell avatars: A_4_2 (A), A_4_5 (B), A_4_6 (C), A_4_7 (D), A_4_8 (E), A_4_28 (F), K_4_143 (G), K_4_161 (H), K_4_207 (I) were tested for their capacity to respond to islet and spleen peptide extracts (diluted 1:200 in media). Positive controls were synthetic HIP peptides. Responses were measured by luciferase assay and represented by Δluciferase, calculated by subtracting the mean of 1:200 dilution of dissolved in dimethyl sulfoxide in media treated samples from the other treatment groups. One representative of two experiments is shown. Statistical significance was determined using a paired Student's t test comparing spleen extract with the islet extract and defined as *P < 0.05, **P < 0.01, ***P < 0.001.

Because identifying a specific HIP species, or set of HIPs, in human islets is very challenging (33, 34), we took a functional approach to demonstrate the relevance of T cell specific for the HIPs we have identified. We tested the capacity of HIP-specific T cell avatars to respond to peptide extracts of human islets and spleen which we used a control tissue. Of the nine avatars tested, six (five from Donor A and one from Donor K) responded strongly to islet, but not to spleen extracts (Figure 3). The remaining three avatars had weaker, but statistically significant, responses to islet-peptide extracts compared to the control spleen peptide extracts. This reveals that the T cell avatars respond to peptides present in human islet, but not those in the spleen.

### HIPs stimulate CD4^+^ T cell response in the peripheral blood of individuals with T1D

**Fig. 4.**
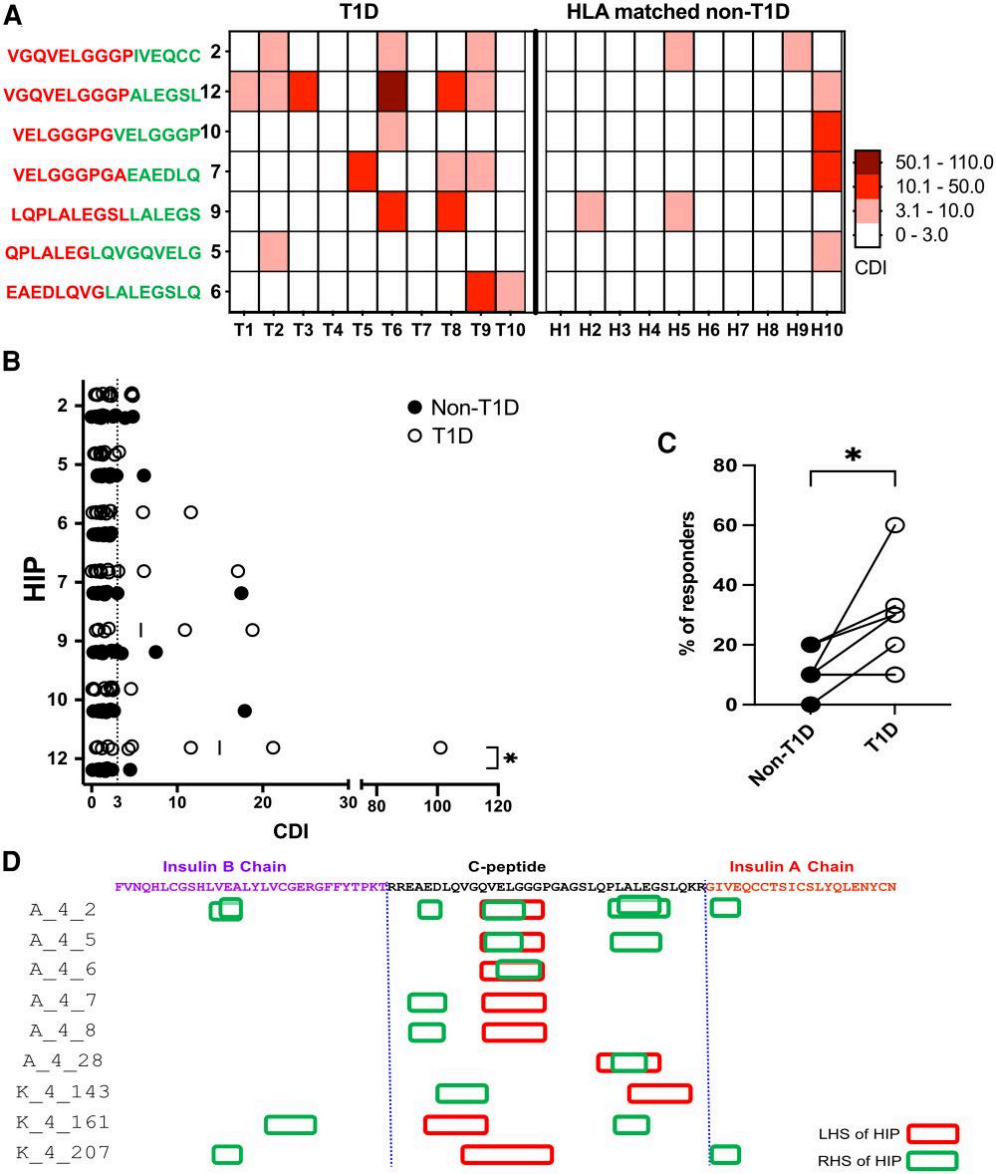
PBMC CD4^+^ T cells from individuals with T1D respond to newly identified HIPs. A) Heat map showing the magnitude of responses to individual HIPs (1 μM), expressed by CDI (35) for PBMC from individuals with T1D and individuals without T1D. A CDI < 3.0, considered to be a negative response, is represented by a white box. The color scale indicates the strength of the CD4^+^ T cell responses to the peptides indicated. “X” indicates not determined. B) The mean CDIs of triplicates for individuals with T1D (*n* = 10) represented by open circle, and without T1D (*n* = 10) HLA matched, represented by closed circle are plotted. Responses with a CDI ≥ 3.0, indicated by the dotted line, are considered to be positive. C) A comparison of the proportion of the 10 subjects, shown in (A) and (B), that have a detectable CD4^+^ T cell response to proinsulin HIPs. Statistical significance was determined using unpaired one-tail nonparametric Mann–Whitney test, *P < 0.05. D) Shows a summary of all the HIPs mapped to the location of each fragment in proinsulin. The sequence of proinsulin is shown in a linear form with each amino acid indicated in single letter code. The C-peptide is bordered by vertical dotted lines. The red boxes indicate the NH_2_-terminal peptide fragment and the green the C-terminal peptide fragment. Each line indicates a different T cell avatar. Avatars from Donor A, start with “A_” and avatars from Donor K start with “K_”. Where a T cell avatar has responded to multiple HIPs there are several boxes shown (for example, avatar A_4_2).

We used the carboxyfluorescein succinimidyl ester (CFSE)-based proliferation assay (35) to determine if the responses to the proinsulin HIPs we had identified were detectable in the PBMCs of 10 individuals with T1D and 10 individuals without T1D who expressed the HLA-DR4-DQ8 haplotype (Tables 7 and 8). Where a T cell avatar responded to several HIPs, the most potent HIP of this family was used (see Table S9) for these experiments. A summary of the PBMC response to seven HIPs is shown in Figure 4A. Overall CD4^+^ T cell response to proinsulin HIPs were more frequently detected in PBMC from individuals with T1D than those without T1D (Figure 4A and B). The magnitude of the response to HIPs was generally greater in the T1D PBMC compared to the non-T1D PBMCs. However, this difference only reached statistical significance for HIP-L. Collectively, the CFSE-based proliferation assay responses to the HIP peptides (cell division index [CDI] > 3.0 (35)) were detected significantly (*P* < 0.05) more frequently in PBMC from individuals with T1D than the HLA-matched non-T1D subjects (Figure 4C). This was most conspicuous for CD4^+^ T cell response to HIP-L which were detected in 60% (6 of 10) of T1D samples, but only 10% (1 of 10) of non-T1D samples (Figure 4A). Response to HIP-F were detected in 20% (2 of 10) of T1D subjects but were not detected in PBMCs from any individuals without T1D (Figure 4A). Hence, we conclude PBMCs from individuals with T1D respond more frequently, and more strongly, to the new HIPs we have identified than the control subjects who do not have T1D.

When the origins of each HIP fragment were mapped against the sequence of proinsulin (Figure 4D). It was clear that C-peptide is a major source of peptide fragments which form HIPs. While some sequences appeared in more than one HIP (Figure 4D), all the N-terminal (left-hand side) peptide fragments that contribute to HIPs originate from C-peptide. For the C-terminal (right-hand side) fragments, 62% (8 of the 13) fragments were derived from C-peptide. In contrast, 23% (3 of 13) C-terminal fragments are derived from B-chain, and 8% (1 of 13) are derived from A-chain of proinsulin. Hence, we conclude that in addition to being an important antigen in its own right (10), proinsulin C-peptide is a dominant source of peptide fragments which fuse to form HIPs that are recognized by human islet-infiltrating CD4^+^ T cells when presented by HLA-DQ8 or HLA-DR4. Interestingly, eight of the nine TCRs that recognized novel proinsulin HIPs expressed TRBV 5-1 or TRBV20-1 (5 and 3, respectively, see Table S10).

## Discussion

This paper is the first to screen a large panel of human TCRs derived from human islet-infiltrating CD4^+^ T cells for reactivity to a large panel of candidate HIPs. Here we report the identification of 13 previously unknown HIPs. These HIPs are all recognized by TCRs derived from CD4^+^ T cells isolated from the pancreatic islets of individuals who had type 1 diabetes. They are restricted by HLA-DQ8 or HLA-DR4 and are formed by the fusion of proinsulin-derived peptides. The CD4^+^ T cell avatars that recognize these HIPs also respond specifically to islet-peptide extracts.

We set out with two goals. First, to make as few assumptions as possible about the identity of candidate novel HIPs. Second, to use our large panel of T cell avatars that express TCRs from human islet-infiltrating CD4^+^ T cells, to identify novel proinsulin-derived HIPs. We reasoned that HIPs recognized by TCRs from human islet-infiltrating CD4^+^ T cells would be very relevant to the immune pathogenesis of human T1D. To achieve this goal, we focused on screening candidate HIPs formed by the fusion of peptides from proinsulin. In total, we screened 109 T cell avatars against our library of candidate HIPs. Surprisingly, all the HIPs we identified incorporated peptides derived C-peptide on the N-terminus (Figure 4D). Of the nine TCR-transduced T cell avatars that responded in our screen, three were previously shown to respond to C-peptide and two had been shown to recognize a C-peptide-IAPP2 HIP (Table S6) (5, 30). However, no agonist epitopes had been identified for four T cell avatars, one from Donor A, and three from Donor K. Consistent with our earlier work (5), the N-terminal component of the HIPs recognized by the Donor A T cell avatars, previously shown to respond to C-peptide-derived epitopes, mapped to the central region of C-peptide. Similar cross-reactivity has been reported in the NOD mouse, where T cells specific for a HIP comprising insulin B-chain cross-react with insulin_B9-23_ (36). Most, but not all the C-terminal sequences also originated from C-peptide (Figure 4D). Two of the HIPs identified here are similar to HIPs reported previously by Delong et al. (15). They reported a Cpept–Cpept HIP, (HIP 3: GQVELGGG-EADELQV) which is similar to our HIP-G(VELGGGPGA-EAEDLQ). In our HIP-G, there are three additional amino acids (PGA) on the N-terminal fragment. The second pair of similar HIPs are Delong's HIP4 (GQVELGGG-GIVEQCC), which is one amino acid different from our HIP-B (GQVELGGGP-IVEQCC), there is effectively a substitution of P for a G at the junction point.

By design, our screen did not include every possible HIP that could be formed in human beta cells because the number of candidate HIPs is enormous, making such a screen impossible (26). Instead, we have limited our screen to HIPs formed by fusion of proinsulin-derived peptides. However, even proinsulin, which is 86 amino acids long, can theoretically generate over 9,400 HIPs. To enrich for potentially immunogenic HIPs, we selected candidate HIPs that were predicted to bind strongly to HLA-DQ8. This selection may have led us to exclude some HIPs which bind relatively weakly to HLA-DQ8, so we do not suggest that we have identified every possible HLA-DQ8 restricted HIP. However, 85% (11 of 13) HIPs specific avatars were HLA-DQ8 restricted while two were HLA-DR4 restricted, suggesting that our screen identified the majority of HLA-DQ8 restricted, proinsulin-derived, HIPs. Nonetheless, both HLA-DQ8 and HLA-DR4 are strongly associated with risk of T1D (32) supporting the relevance of these TCRs and the HIPs they recognize to the immune pathogenesis of T1D. In this study, we did not attempt to identify HIPs restricted by other T1D-associated HLA allomorphs; such as: HLA-DR4, DR3, DQ2, or DQ2/8transdimers.

The reason for strong representation of C-peptide in HIPs is not clear, but several factors may contribute. C-peptide is abundant in the beta cells and unstructured (37–39). Des-(27-31) C-peptide, a product of cathepsin D mediated C-peptide cleavage, is also relatively abundant in islets and may contribute to HIP formation (40–42). The protease cathepsin D has broad specificity (43) allowing it to cleave C-peptide are several positions, which is consistent with our findings that there is no clear motif at the points of peptide fusion. Spontaneous formation of HIPs via an aspartate anhydride intermediate has also been proposed as a mechanism for HIP formation (44). However, the HIPs we have identified do not appear to be formed by this mechanism because they do not have the EAED sequence at the N-terminal side of the fusion point.

TCR sequencing studies have allowed the analysis of TCR usage in T1D. Strikingly, the HIPs specific TCRs that we identified here were limited in TRBV usage (Table S10). Almost all used TRBV 5-1, or TRBV20-1. This extends our observation that these TRBV alleles are commonly expressed by human islet-infiltrating CD4^+^ T cells specific for C-peptide and other HIPs (5, 30). The structure of TCR/pHLA complexes for three TCRs (A_4_2, A_4_5, and A_4_6) have been solved. The N- and C-termini of the HIP interact with TRA and TRB chains, respectively. This work revealed that the TRBV5-1 allele can form a “landing pad” on HLA-DQ8 which may, in part at least, explain is over-representation in HIP-specific TCRs. Further work is required to determine the crystal structure of the proinsulin HIP/HLA/TCR complexes identified here to determine, at the molecular level, how TCRs recognize multiple HIPs. Recent advances in the analysis of TRBV sequences in samples from individuals with and without T1D suggest that TCR analysis may eventually be applied to the monitoring T cell responses in a clinical setting.

When we examined PBMC responses to the newly identified HIPs, we found that each individual with T1D has a CD4^+^ T cell response to a unique set of HIPs detected using the CFSE-based proliferation assay (35). We detected CD4^+^ T cell responses to one or more HIPs in PBMC from 20% (2 of 10) of non-T1D individuals and from 80% (8 of 10) of individuals with T1D. We found that some HIPs more frequently stimulate T cell responses, but no “dominant” HIP has been identified (Figure 4A). This is in concordance with other reports which have examined PBMC responses to HIPs in individuals with and without T1D (21, 23, 45). Collectively, this suggests that if HIPs are to be applied to the analysis of T cell responses, or in an antigen-specific therapy, a carefully curated cocktail of HIPs may be required, or perhaps a personalized combination of HIPs.

There are some limitations of our study. We could only study TCRs derived from four T1D organ donors which had widely varying durations of T1D. It is becoming clear that beta cell antigen-specific responses become more difficult to detect as time since diagnosis with stage 3 T1D increases. Although this time since diagnosis effect is difficult to rigorously demonstrate, without a longitudinal study, we have observed it in previous experiments. However, we cannot exclude the possibility that HIP-specific CD4^+^ T cells are present in the islets of donors with longer-duration T1D, despite our failure to detect any in this study. As noted above, our screen was limited to HIPs formed by fusion of proinsulin peptide fragments. Clearly, many HIPs are formed by fusion of proinsulin peptides with peptides from other protein (6, 11, 15). However, in this study, we have not attempted to identify these “non-proinsulin” HIPs. Based on our data, we cannot comment on whether peptides derived from C-peptide are common components of HIPs formed from other beta cell proteins. Similar to others (15, 23, 30, 31, 46), we found that that some TCRs respond to several HIPs and in some cases cross-reacted weakly with native C-peptide. T cell responses were seen to families of closely related HIPs (Figure 2J-M). While these are different sequences they have many residues in common. Both the N-terminal part of the HIP and the putative anchor residues are conserved in all cases. Each family of HIPs only differs in one to three residues within the core 9mer. Given that TCRs have some flexibility (30), Tran, 2024 #6449], this degree of cross-reactivity is perhaps not surprising. At this point, it is not clear if all these related HIPs form in vivo and are targets of CD4^+^ T cell responses, or if only some of them form. Our data (Figure 4), and data from others (21), suggest that each individual may respond to a unique set of HIPs. It should be noted that we cannot rigorously exclude the possibility that the proinsulin HIPs we have identified are mimotopes which mimic the sequence of the “real” HIP which forms in vivo. However, we believe this is very unlikely because: proinsulin is a critical antigen in human T1D (8), the HIPs we have identified are recognized by TCRs derived from human islet-infiltrating CD4^+^ T cells, and these TCRs also respond specifically to human islet extracts (Figure 3). We show that our HIP-specific T cell avatars also respond to a peptide extract from human islets. These T cell avatars very sensitive to the proinsulin HIPs we have identified, but they did not respond to C-peptide, except in the three cases where we saw very weak responses at the highest concentrations tested (20 μM). This suggests that the HIPs we have identified are present in human islets, but we have not specifically defined these proinsulin HIPs as the agonist species in these extracts.

## Conclusion

In conclusion, using T cell lines expressing TCR pairs derived from human islets we screened a library of all possible HIPs formed by the fusion of proinsulin peptides. We identified 13 new proinsulin-derived HIPs. All HIPs incorporated fragments of C-peptide, but rarely peptides from insulin A or B chains. In addition to confirming the clinical relevance of CD4^+^ T cell response to HIPs in human T1D, this work shows that C-peptide is a major source of HIPs recognized by human islet-infiltrating CD4^+^ T cells.

## Supplementary Material

pgae491_Supplementary_Data

## Data Availability

The TRAV and TRBV sequences of all TCRs used in this study are available on OSFHOME database at: https://osf.io/DOI 10.17605/OSF.IO/QG23Z.

## Preprints

This manuscript was posted on a preprint: https://doi.org/10.1101/2024.05.09.593303.
