# Supplementary material for: Proinsulin C-peptide is a major source of HLA-DQ8 restricted hybrid insulin peptides recognized by human islet-infiltrating CD4+ T cells
Source: PNAS Nexus. 2024 Nov 1;3(11):pgae491. doi: 10.1093/pnasnexus/pgae491 (PMC11565411; doi:10.1093/pnasnexus/pgae491)
Supplement: pgae491_Supplementary_Data [file pgae491_supplementary_data.docx]

**Proinsulin C-peptide is a major source of HLA-DQ8 restricted HIPs recognized by human Islet-Infiltrating CD4^+^ T cells**

Pushpak Bhattacharjee^a^, Miha Pakusch^a^, Matthew Lacorcia, Eleonora Tresoldi^a^, Alan F. Rubin^f,g^, Abby Foster^a^, Laura King^a^, Chris Chiu^a^, Thomas W.H. Kay^a^, John A. Karas^e^, Fergus J. Cameron^c,d^, Stuart I. Mannering ^a,b,d*^

**Supplementary Tables**

Supplementary Table 1: Primers used in this study.

Supplementary Table 2: Peptides used in this study

Supplementary Table 3: Demographics and HLA of organ donors.

Supplementary Table 4: Optimization of the bacterial epitope mapping (BEM) system.

Supplementary Table 5. Summary of avatars screened and new HIPs identified.

Supplementary Table 6: Summary of previously characterised islet-infiltrating CD4^+^ T cells.

Supplementary Table 7: Demographics and HLA of T1D PBMC donors.

Supplementary Table 8: Demographics and HLA of non-T1D PBMC donors.

Supplementary Table 9: Summary of characteristics of the newly identified HIPs

Supplementary Table 10: Summary HIP specific TCRs

Supplementary Table 11: New HIPs and their predicted HLA-DQ8 binding rank

**Supplementary Figures**

Supplementary Figure 1: Generation of Jurkat T-cell avatars

Supplementary Figure 2: Overview of the generation of candidate proinsulin HIPs for screening Supplementary Figure 3: T-cell avatars barely respond to native N terminal or C-terminal proinsulin peptide but are very sensitive to HIPs derive from proinsulin.

Supplementary Figure 4: Most HIP specific CD4^+^ T-cell responses are restricted by HLA-DQ8

**Supplementary Table 1:** List of primers used in this study.

| **Primer name** | **Sequence** |
| --- | --- |
| HIP Pool Forward Primer | GATCTGGTTCCGCGTGG |
| HIP Pool Reverse Primer | GTCGATGCCAGGATCGG |
| Amplicon Forward Primer | AATCGGATCTGGTTCCGC |
| Amplicon Reverse Primer | AGTCGATGCCAGGATCGG |
| pKE-1 sequencing Primer | CGTATTGAAGCTATCCCAC |

**Supplementary Table 2:** Peptides used in this study.

| **Peptide name** | **Peptide sequence** |
| --- | --- |
| HIP-A | VGQVELGGG-LALEGSL |
| HIP-B | VGQVELGGGP-IVEQCC |
| HIP-C | VGQVELGGGP-LVEALY |
| HIP-D | VGQVELGGGP-GVEALY |
| HIP-E | VGQVELGGGP-ALEGSL |
| HIP-F | VELGGGPG-VELGGGP |
| HIP-G | VELGGGPGA-EAEDLQ |
| HIP-H | GQVELGGGPGA-EDLQ |
| HIP-I | LQPLALEGSL-LALEGS |
| HIP-J | QPLALEG-LQVGQVELG |
| HIP-K | REAEDLQVG-VCGERGF |
| HIP-L | EAEDLQVG-LALEGSLQ |
| HIP-M | RREAEDLQVGQ-ALEGS |
| HIP-6 | GQVELGGG-NAVEVLK |
| C-peptide | EAEDLQVGQVELGGGPGAGSLQPLALEGSLQ |
| PPI Pep# 9 | FYTPKTRREAEDLQVGQV |
| PPI Pep# 10 | RREAEDLQVGQVELGGGP |
| PPI Pep# 11 | LQVGQVELGGGPGAGSLQ |
| PPI Pep# 13 | GAGSLQPLALEGSLQKRG |
| PPI Pep# 15 | SLQKRGIVEQCCTSICSL |

**Supplementary Table 3**: Demographics and HLA detail of organ donors

| **SVI identifier** | **Study Identifier (Sex)** | **T1D duration**  **(years)** | **Age** | **No. avatars** | **HLA Class I** | **HLA Class II** |
| --- | --- | --- | --- | --- | --- | --- |
|  |  |  | **(years)** |  |  |  |
| SVI021-08 | A (M) | 3 | 19 | 39 | A 01:01, 02:01  B 08:01, 51:01  C 07:01, 15:02 | DRB1*03:01, 04:04  DPB1*01:01, 04:01/ DPA1*01:03, 02:01  DQB1*02:01/163N, 03:02; DQA1*03:01, 05:01 |
| SVI-012-19 | I (F) | 13 | 59 | 10 | A 01:01, 02:01  B 08:01, 15:01  C 03:03, 07:01 | DRB1*03:01, 04:01  DPB1*04:01, 20:01/ DPA1*01:03  DQB1*02:01/163N, 03:02/289 DQA1*03:01, 05:01 |
| SVI-013-19 | J (M) | 19 | 54 | 8 | A 03:01, 68:01  B 07:02, 40:01  C 03:04, 07:02 | DRB1*01:03, 04:01  DPB1*02:01, 04:01/ DPA1*01:03  DQB1*03:02/289, 05:01 DQA1*01:01, 03:01 |
| SVI-017-21 | K (F) | 4 | 13 | 52 | A02; 68  B*08, 15:01  C*03:03, 07 | DRB3*01, DRB4*01  DPB1*01:01, *10:01/DPA1*02  DQB1*02, 03:02  DQA1*03, 05 |

**Supplementary Table 4**: Optimization of the bacterial epitope mapping (BEM) system.

| **Optimizing parameter** | **Parameter tested** | **Optimal condition** |
| --- | --- | --- |
| Bacterial Strain | Stellar, NEB, Rosetta, DH5a | Rosetta |
| Bacterial load | 10 and 20 ml | 10 ml |
| Bacterial dilution | Neat to 1:2,560 | 1:150 |
| IPTG conc. | 0 - 16mM | 2 mM |
| Storage condition | Glycerol stock, patch plate & fresh | No effect |
| Complement | With and without | No effect |
| Temp for o/n bug culture | 30^0^C and 37^0^C | 37^0^C |
| Length of the epitope | 12mer, 15mer & 24mer | 12mer |
| Flanking the epitope | 1 and 2 GSSS linkers | No linker |
| Protease site | Cat S site at both side of epitope | Not required |

**Supplementary Table 5.** Summary table of the numbers of avatars screened and new HIPs identified.

| **Donor** | **Avatars**  **Screened** | **Avatars that**  **responded HIP** | **Neo-HIP Identified** |
| --- | --- | --- | --- |
| A | 39 | 6 | 9 |
| I | 10 | 0 | 0 |
| J | 8 | 0 | 0 |
| K | 52 | 3 | 6 |
| **Total** | **109** | **9** | **13** |

**Supplementary Table 6.** Summary of previous characterisation of human islet-infiltrating CD4^+^ T cells.

| **Current nomenclature** | **Previous nomenclature** | **Epitope** | **HLA-restriction** | **Reference** |
| --- | --- | --- | --- | --- |
| A_4_2 | A1.9 | VELGGGPGA | HLA-DQ8 | [5, 34] |
| A_4_5 | A4.5 | GQVELGGG-NAVEVLK | HLA-DQ8 | [15, 34] |
| A_4_6 | A3.10 | GQVELGGG-NAVEVLK | HLA-DQ8 | [15, 34] |
| A_4_7 | A5.5 | VELGGGPGA | HLA-DQ8 | [5] |
| A_4_8 | A5.8 | VELGGGPGA | HLA-DQ8 | [5] |

ND- not previously determined.

Not applicable

HIP sequ-ences

**Supplementary Table 7**. Demographics and HLA of T1D PBMC donor

| **SVI Identifier** | **Identifier (Sex)** | **T1D Duration (years)** | **Age at sampling (year)** | **HLA Class I** | **HLA Class II** |  |
| --- | --- | --- | --- | --- | --- | --- |
| 624T1 | T1 (F) | 11 | 18 | A 02:01, 30:02  B 18:01,44:02  C 05:01, 05:01 | DRB1*03:01, 04:01  DQA1*03:03, 05:01/DQB1*02:01, 03:01  DPB1*03:01, 04:01 |  |
|  |  |  |  |  |  |  |
|  |  |  |  |  |  |  |
| 625T1 | T2 (F) | 4 | 11 | A 02:01  B 07:02, 40:01  C 03:04, 07:02 | DRB1*03:01, 04:04  DQA1*03:01, 05:01/DQB1*02:01, 03:02  DPB1*02:01, 04:01 |  |
|  |  |  |  |  |  |  |
|  |  |  |  |  |  |  |
| 626T1 | T3 (F) | 3 | 10 | A 02:01, 26:01  B 08:01,40:23  C 03:04, 07:02 | DRB1*03:01, 09:01  DQA1*03:02, 05:01/DQB1*02:01, 03:02  DPB1*04:01, 02:01 |  |
|  |  |  |  |  |  |  |
|  |  |  |  |  |  |  |
| 627T1 | T4 (F) | 8 | 17 | A 11:01, 30:01  B 13:02, 40:01  C 03:04, 06:02 | DRB1*04:01, 04:04  DQA1*03:01/DQB1*03:02, 03:02  DPB1*03:01, 16:01 |  |
|  |  |  |  |  |  |  |
|  |  |  |  |  |  |  |
| 631T4 | T5 (M) | 64 | 68 | A 23:01, 29:02  B 08:01, 44:03  C 07:01, 16:01 | DRB1*03:01, 04:04  DQA1*03:01, 05:01/DQB1*02:01, 03:02  DPB1*04:01, 15:01 |  |
|  |  |  |  |  |  |  |
|  |  |  |  |  |  |  |
| 632T1 | T6 (M) | 6 | 7 | A 02:01  B 40:01, 40:01  C 03:04, 03:04 | DRB1*09:01  DQA1*03:02, /DQB1*03:03, 03:03  DPB1*05:01 |  |
|  |  |  |  |  |  |  |
|  |  |  |  |  |  |  |
| 642T1 | T7 (F) | 0.1 | 9 | A 02:01, 68:01  B 15:01, 44:02  C 03:03, 05:01 | DRB1*04:01, 04:04  DQA1*03:01/DQB1*03:02, 03:02  DPB1*04:01, 06:01 |  |
|  |  |  |  |  |  |  |
|  |  |  |  |  |  |  |
| 651T1 | T8 (M) | 7 | 16 | A 03:01, 23:01  B 07:02,13:02  C 06:02, 07:02 | DRB1*04:01, 11:01  DQA1*03:01, 05:05/DQB1*03:01, 03:02  DPB1*02:01, 04:01 |  |
|  |  |  |  |  |  |  |
|  |  |  |  |  |  |  |
| 652T1 | T9 (M) | 10 | 16 | A 03:01, 68:01  B 08:01, 35:01  C 04:01, 07:01 | DRB1*03:01, 04:01  DQA1*03:01, 05:01/DQB1*02:01, 03:02  DPB1*02:01, 04:01 |  |
|  |  |  |  |  |  |  |
|  |  |  |  |  |  |  |
| 653T1 | T10 (M) | 3 | 6 | A 01:03, 03:01  B 07:02, 07:02  C 07:02, 07:02 | DRB1*03:01  DQA1*05:01/DQB1*02:01, 02:01  DPB1*02:01,665:01 |  |
|  |  |  |  |  |  |  |
|  |  |  |  |  |  |  |

**Supplementary Table 8:** Demographics and HLA of non-T1D PBMC donors

| **Donor** | **Identifier (Sex)** | **Age at donation (years)** | **HLA Class I** | **HLA Class II** |
| --- | --- | --- | --- | --- |
| 138H4 | H1 (F) | 50 | A 11:01, 31:01  B13:01, 35:01  C 03:03, 03:04 | DRB1*04:03, 06:02  DQA1*01:02, 03:01/DQB1*03:02, 05:02  DPB1*13:01 |
| 26H2 | H2 (M) | 45 | A 11:01, 24:02  B35:03, 40:01  C03:04, 12:03 | DRB1*04:01,04:08  DQA1*03:01,03:03 /DQB1*03:02, 03:04  DPB1*02:01, 15:01 |
| 32H2 | H3 (F) | 42 | A 11:01, 24:02  B39:01, 55:02  C03:03, 07:02 | DRB1*04:03, 14:54  DQA1*01:04, 03:01/DQB1*03:02, 05:02  DPB1*05:01 |
| 133H4 | H4 (F) | 31 | A 11:01, 29:02  B40:01, 51:01  C03:04, 14:02 | DRB1*04:04, 11:01  DQA1*03:01, 05:05/DQB1*03:01, 03:02  DPB1*04:01, 14:01 |
| 73H4 | H5 (M) | 36 | A 01:01, 02:01  B08:01, 15:01  C03:03, 07:01 | DRB1*03:01, 04:01  DQA1*03:01, 05:01/DQB1*02:01, 03:02  DPB1*04:01 |
| 368H4 | H6 (M) | 22 | A 01:01, 03:02  B35:03, 40:01  C03:04, 04:01 | DRB1*04:03, 13:03  DQA1*03:01,05:05 /DQB1*03:01, 03:02  DPB1*02:01 |
| 33H2 | H7 (F) | 51 | A 02:01, 24:02  B 15:01, 51:01  C 03:03, 14:02 | DRB1*04:01, 14:03  DQA1*03:01, 05:07/DQB1*03:01, 03:02  DPB1*03:01, 05:01 |
| 294H4 | H8 (F) | 25 | A 02:01, 11:01  B 44:470, 56:01  C 01:02, 05:01 | DRB1*04:39, 13:01  DQA1*01:03, 03:01/DQB1*03:02, 06:03  DPB1*04:01, 14:01 |
| 14H2 | H9 (M) | 35 | A 01:01, 01:01  B 08:01, 37:01  C 06:02, 07:01 | DRB1*03:01, 10:01  DQA1*05:01, 01:05/DQB1*02:01, 02:163N, 02:198  DPB1*01:01, 02:01 |
| 275H4 | H10 (M) | 21 | A 02:01, 26:01  B 27:05, 51:01  C 01:02, 07:02 | DRB1*01:01, 04:03  DQA1*01:01, 03:01/DQB1*03:02, 05:01  DPB1*04:01, 13:01 |

**Table 9:** Summary of characteristics of the newly identified HIPs.

| **HIP** | **Sequence** | **T Cell Avatar** | **EC50 (μM)** | **HLA restriction** |
| --- | --- | --- | --- | --- |
| A | VGQVELGGG-LALEGSL | A_4_2 | 13.89 | DQ8 |
|  |  | A_4_5 | 2.49 |  |
| B * | VGQVELGGGP-IVEQCC | A_4_2 | 1.66 | DQ8 |
|  |  | K_4_207 | 2.81 |  |
| C | VGQVELGGGP-LVEALY | A_4_2 | 10.69 | DQ8 |
| D | VGQVELGGGP-GVEALY | A_4_2 | 6.12 | DQ8 |
|  |  | K_4_207 | 8.27 |  |
| E * | VGQVELGGGP-ALEGSL | A_4_2 | 1.88 | DQ8 |
|  |  | A_4_5 | 1.61 |  |
| F * | VELGGGPG-VELGGGP | A_4_2 | 3.24 | DQ8 |
|  |  | A_4_6 | 5.18 |  |
|  |  | A_4_5 | 10.27 |  |
| G* | VELGGGPGA-EAEDLQ | A_4_2 | 2.40 | DQ8 |
|  |  | A_4_8 | 0.43 |  |
|  |  | A_4_7 | 0.57 |  |
| H | GQVELGGGPGA-EDLQ | A_4_2 | 5.81 | DQ8 |
| I * | LQPLALEGSL-LALEGS | A_4_28 | 0.02 | DR4 |
| J * | QPLALEG-LQVGQVELG | K_4_143 | 0.32 | DR4 |
| K | REAEDLQVG-VCGERGF | K_4_161 | >20.00 | DQ8 |
| L* | EAEDLQVG-LALEGSLQ | K_4_161 | 0.18 | DQ8 |
| M | RREAEDLQVGQ-ALEGS | K_4_161 | 2.78 | DQ8 |

*Represents HIPs used for CFSE assay

**Supplementary Table 10.** Summary HIP specific TCRs

| **Cell line ID** | **TRAV** | **TRAJ** | **tra_cdr3** | **TRBV** | **TRBD** | **TRBJ** | **trb_cdr3** |
| --- | --- | --- | --- | --- | --- | --- | --- |
| A_4_2 | 20*02/*04 | 7*01 | CAVQAGGNNRLAF | 5-1*01 | 1*01 | 1-2*01 | CASSLERDGYTF |
| A_4_5 | 38-1*03 | 54*01 | CAFMGAGAQKLVF | 4-3*01 | 2*01 | 2-3*01 | CASSQILRGGPPDTQYF |
| A_4_6 | 38-1*03 | 54*01 | CAFFGQGAQKLVF | 5-1*01 | 2*01 | 2-3*01 | CASSLSASGGATDTQYF |
| A_4_7 | 26-1*01 | 39*01 | CIVSHNAGNMLTF | 5-1*01 | 2*01 | 2-5*01 | CASSLERETQYF |
| A_4_8 | 26-1*02 | 21*01 | CIAIYNFNKFYF | 5-1*01 | 2*01 | 1-6*02 | CASSLEASSYNSPLHF |
| A_4_28 | 36/DV7*04 | 53*01 | CATRRGSNYKLTF | 20-1*01 | 2*01 | 2-3*01 | CSAPLSGGSTDTQYF |
| K_4_143 | 4*01 | 36*01 | CLVGDAGTGANNLFF | 20-1*01 | 1*01 | 2-7*01 | CSAREVGTVSYEQYF |
| K_4_161 | 9-2*03 | 5*01 | CALSDMGRRALTF | 20-1*01 | 1*01 | 2-1*01 | CSASIGQLSSYNEQFF |
| K_4_207 | 26-1*01 | 54*01 | CIVRVEIQGAQKLVF | 5-1*01 | 2*02 | 2-5*01 | CASSLGPGARETQYF |

**Supplementary Table 11.** New HIPs and their predicted HLA-DQ8 binding rank

| **HIP** | **Sequence** | **HLA-DQ8 predicted binding rank^** |
| --- | --- | --- |
| A | VGQVELGGG-LALEGSL | 7.0 |
| B | VGQVELGGGP-IVEQCC | 6.0 |
| C | VGQVELGGGP-LVEALY | 5.6 |
| D | VGQVELGGGP-GVEALY | 19.0 |
| E | VGQVELGGGP-ALEGSL | 9.4 |
| F | VELGGGPG-VELGGGP | 12.0 |
| G | VELGGGPGA-EAEDLQ | 7.5 |
| H | GQVELGGGPGA-EDLQ | 15.0 |
| I | LQPLALEGSL-LALEGS | 9.0 |
| J | QPLALEG-LQVGQVELG | 2.1 |
| K | REAEDLQVG-VCGERGF | 4.4 |
| L | EAEDLQVG-LALEGSLQ | 0.2 |
| M | RREAEDLQVGQ-ALEGS | 0.4 |

^When the putative peptide had multiple HLA binding registers, the highest (i.e lowest % rank) is reported.

**Supplementary Figure 1:** *Generation of Jurkat T-cell avatars.*

The Jurkat line, Jurkat E6.1, was modified using CRISPR/Cas9 to delete the endogenous CD4, TRA and TRB genes. These cells were cloned and clone 2.2 was selected. Next, Nanoluciferase was knocked into the IL-2 locus and the cells containing the knock-in construct were cloned. From this clone 3.1 was selected. Finally, this clone was transduced with human CD4^+^ and TRA and TRB constructs with TCR sequences from human islet-infiltrating CD4^+^ T-cell clones. These T-cell avatars were then used to screen the bacterial library for HIPs that they recognized.

**
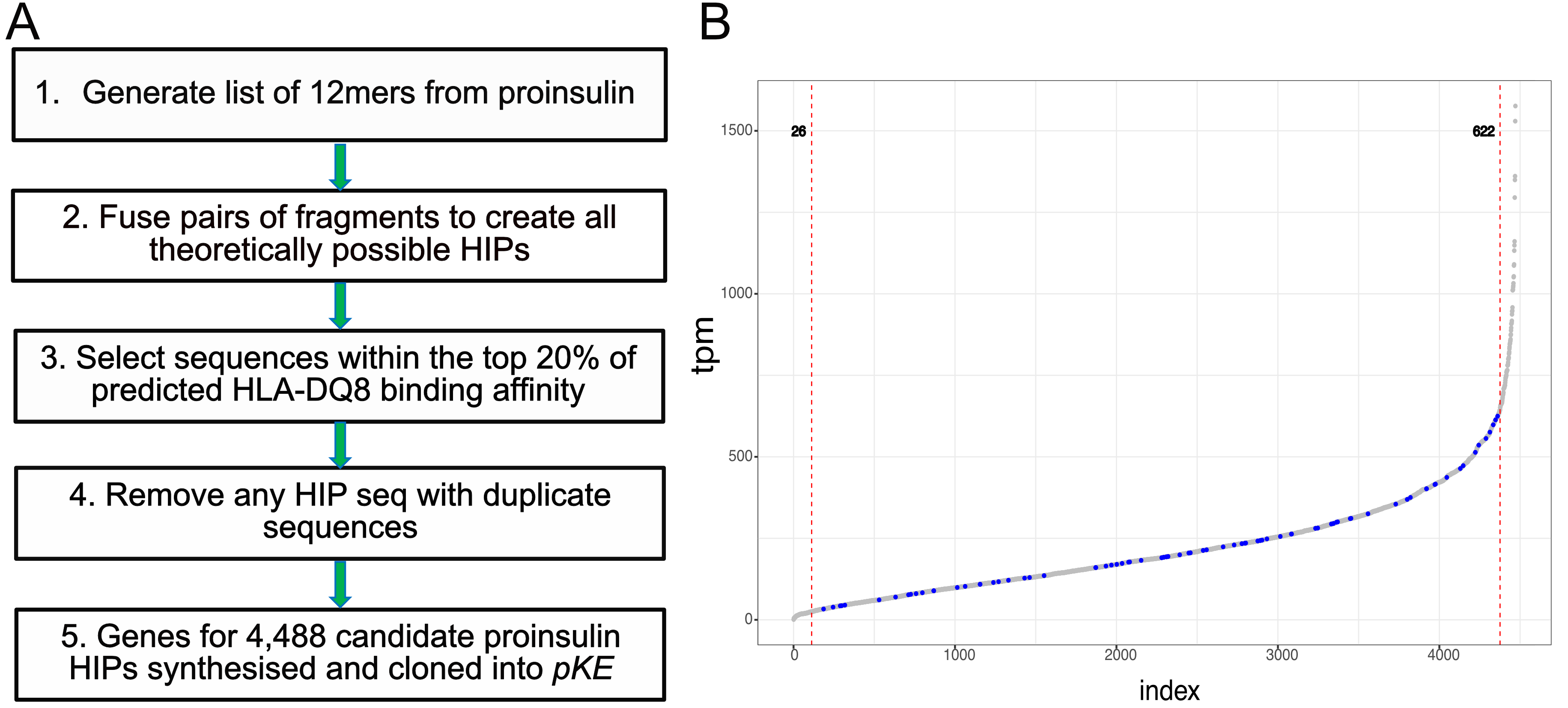
**

**Supplementary Figure 2:** *Overview of the generation of candidate proinsulin HIPs for screening.* (A) *In silico* generation of the proinsulin candidate HIP library. (B) Sequencing of the master HIP library comprising 4,488 candidate HIPs. The dotted vertical red lines contain 95% of the distribution. Blue dots represent HIPs that were identified by screening with T-cell avatars expressing TCRs derived from human islet-infiltrating CD4^+^ T-cell clones.

**Supplementary Figure 3:** *T-cell avatars barely respond to native N terminal or C-terminal proinsulin peptide but are very sensitive to HIPs derive from proinsulin*. Dose-response curves for the following T-cell avatars are shown: (A) A_4_2 tested against HIP-B (circles), N-terminal-side PPI #11(squares) and C-terminal PPI #15(triangle); (B) T-cell avatar A_4_5 was tested against HIP-A (circle), N-terminal PPI #11 (squares) and C-terminal PPI #13 (triangle). (C) T-cell avatar A_4_6 was tested against HIP-F ( circle), N-terminal and right sequence is the same PPI #11 (squares) (D) T-cell avatar A_4_7 was tested against HIP-G (circle), N-terminal PPI #11 (squares) and right side PPI #9 (triangle); (E) T-cell avatar A_4_8 was tested against HIP-G (circle), N-terminal PPI #11 (squares) and right side PPI #9 (triangle); (F) T-cell avatar A_4_28 was tested against HIP-I (circle), N-terminal and right sequence is the same PPI #13 (squares). (G) T-cell avatar K_4_143 was tested against HIP-J (circle), N-terminal PPI #13 (squares) and right side PPI #10 (triangle); (H) T-cell avatar K_4_161 was tested against HIP-L (circles), N-terminal PPI #10 (squares) and right side PPI #13 (triangle); (I) T-cell avatar K_4_207 tested against HIP-B (circles), N-terminal PPI #11 (squares) and right side PPI #15(triangle). One representative of at least two experiments is shown. Statistical significance was determined using a paired Student’s t test considering *p<0.05, **p<0.01, ***p<0.001.

**Supplementary Figure 4.** *Most HIP specific CD4^+^ T-cell responses are restricted by HLA-DQ8*.

The HLA-restriction was determined in two steps. First, blocking antibodies against HLA-DR, -DP, or DQ were used to determine the isotype of the restricting HLA (Left hand column). Second, the restricting allele was defined using B cell lines expressing individual HLA molecules (Right hand side column). Where a T-cell avatar responded to more than one HIP, the most potent was used for this analysis. All peptides were used at (1.0μM). For antibody blocking experiments (LHS) the following mAbs were used: anti-HLA-DQ (clone SPV-L3, 1.0μg/mL), anti-HLA-DP (clone B7/21) or anti-HLA-DR (clone L243). To define the restricting allele , B-cell lines, expressing either no HLA ( HLA class II negative T2 cells), or HLA-DQ8*cis* (HLA-DQA1*03:01, HLA-DQB1*03:02), or HLA-DQ2*cis* (HLA-DQA1*05:01; HLA-DQB1*02:01), or HLA-DQ8*trans* (HLA-DQA1*05:01, HLA-DQB1*03:02), or HLA-DQ2*trans* (HLA-DQA1*03:01; HLA-DQB1*02:01). For HLA-DR restricted TCRs, autologous B-cell lines were used as positive controls and DR3-DQ2 (IHW09022) or DR4-DQ8 (IHW09031) homozygous lines were tested. Data for the following avatars is shown: (A) A_4_2, (B) A_4_5, (C) A_4_6, (D) A_4_7, (E) A_4_8, (F) A_4_28, (G) K_4_143, (H) K_4_161, (I) K_4_207 Responses were measured by luciferase assay and represented by Δluciferase, calculated by subtracting the mean of ‘no antigen’ or /and ‘no antibody’ treated samples from the other treatment groups. The bars indicated the mean of triplicate Δluciferase +/- SD. One representative of two experiments is shown. Statistical significance was determined using one-way ANOVA and corrected for multiple comparisons using Dunnett statistical hypothesis testing considering no HLA antibody or no class II HLA as control and defined as *p<0.05, **p<0.01, ***p<0.001, ****p<0.0001.
